# Autoinflammatory Reaction in Dogs Treated for Cancer via G6PD Inhibition

**DOI:** 10.1155/2017/4275305

**Published:** 2017-08-08

**Authors:** Jonathan W. Nyce

**Affiliations:** Advanced Canine Genetic Testing, 399 Arcola Road, P.O. Box 26219, Collegeville, PA 19426-3998, USA

## Abstract

Glucose-6-phosphate dehydrogenase (G6PD) is an oncoprotein that is overexpressed in cancer cells to provide the NADPH required for their increased anabolism. NADPH, sourced from G6PD fuels nucleotide biosynthesis, maintains redox potential of thioredoxin and glutathione and drives the mevalonate pathway that powers many of the basic mechanisms by which cancer cells escape host control. G6PD is thus a target for cancer treatment being addressed by many groups around the world. We have discovered that systemic inhibition of G6PD by high dose dehydroepiandrosterone (DHEA) causes a severe autoinflammatory response in dogs, which does not occur in mice or rats. Since dogs more closely model the human adrenal androgen system than do common laboratory animals, this finding is relevant to the design of G6PD-inhibiting drugs for humans. The autoinflammatory reaction observed closely resembles mevalonate kinase deficiency (MKD), a rare autosomal recessive disease in humans characterized by recurrent febrile attacks, arthralgia, skin rash, and aphthous ulcers of mucocutaneous tissues. In a manner comparable to animal models of MKD, the reconstitution of protein geranylgeranylation blocked the autoinflammatory reaction caused by systemic G6PD inhibition. This autoinflammatory response to systemic G6PD inhibition represents an unexpected result that must be taken into consideration when targeting this oncoprotein.

## 1. Introduction

G6PD, and the pentose phosphate pathway (PPP) for which it serves as gate keeper, represents a principal generator of the NADPH needed for both biomass production and protection from Reactive Oxygen Species (ROS). The central role that G6PD plays in the neoplastic process is evident from the finding that all that is necessary to transform a nontumorigenic cell into one capable of forming tumors* in vivo* is to insert additional active copies of G6PD into it [1]. NADPH is required for reductive biosynthesis of nucleotides, amino acids, and lipids; for the methyl donors required for epigenetic modification of nucleic acids and proteins; for the synthesis of redox selenoproteins; for the dolichol-mediated N-linked glycosylation of the Insulin-like Growth Factor 1 Receptor (IGF1R); and for maintaining glutathione and thioredoxin in the reduced state necessary to detoxify ROS. Under normal circumstances G6PD is tightly regulated. One of its principal regulators is the p53 tumor suppressor protein, which prevents the formation of the active dimer form of G6PD [2]. The p53 tumor suppressor gene is the most frequently mutated gene in cancer, and inactivation of p53 is thought to play a causative role in at least half of all cancers. Notably, mutant p53 enzymes lose their ability to inhibit formation of active G6PD dimers [2]. When p53 loses its ability to regulate G6PD, the increased NADPH that is produced fuels the nucleotide synthesis, biomass production, and enhanced ability to detoxify ROS that are hallmarks of the neoplastic cell.

Dehydroepiandrosterone (DHEA) is a natural, uncompetitive inhibitor of G6PD. The uncompetitive enzyme kinetics of DHEA's inhibition of G6PD is extremely rare in nature because it can produce a feedforward effect dramatically potentiating the level of enzyme inhibition [3]. Since complete loss of G6PD activity is not survivable for a cell [4], the uncompetitive kinetics of DHEA may represent the components of a “kill-switch” that prevents tumorigenesis in cells that have undergone p53 inactivation. Within this scenario, tumors would represent instances in which this “kill-switch” mechanism had malfunctioned in one way or another [5].

We reasoned that there might exist subpopulations of tumors in which this kill-switch had not been completely triggered, but which still might be triggered by administration of DHEA sufficient to inhibit tumor G6PD. In a pilot study of canine cardiac hemangiosarcoma (CH), a canine tumor that has heretofore been rapidly fatal, G6PD inhibition by DHEA resulted in dramatic tumor regression and increase in lifespan [5]. The dramatically improved survival data observed with G6PD inhibition support the concept that G6PD is a druggable target with consequence in cancer chemotherapy.

However, at doses of DHEA capable of inducing tumor regression* in vivo*, we observed a severe autoinflammatory reaction occurring in treated dogs. This autoinflammatory response included recurrent fever, skin rash, aphthous ulcers of mucocutaneous tissues, apparent increases in arthralgia, and ocular involvement. In previous* in vitro* work we had demonstrated that G6PD inhibition by DHEA repressed the mevalonate pathway by depleting the NADPH required for synthesis of isoprene products of this pathway [6]. We also demonstrated that aspects of this inhibition, for example, protein isoprenylation and geranylation, could be reversed by replenishment of depleted isoprene precursors [7]. In the present study, therefore, along with a description of the autoinflammatory reaction, we also sought to determine if replenishment of isoprene precursors would eliminate the autoinflammatory reaction observed in dogs receiving high dose DHEA for cancer.

## 2. Case Histories

*Case 1* was a 28 kg, 11-year-old, neutered male Border Collie that presented with malaise, inappetence, and external signs of internal bleeding. Ultrasonography demonstrated a large renal mass and evidence of metastatic spread to the lungs. A biopsy of the renal mass was performed, and histological examination revealed sarcoma of endothelial origin.

Under the care of a licensed veterinarian, this dog was entered into our research protocol employing high dose oral DHEA (60 mg/kg/day) and ubiquinone (0.1 mg/kg/day), in divided daily doses. A dramatic improvement in quality of life (appetite, playfulness) occurred within a few days of initiation of the protocol. The patient was reexamined at biweekly intervals by his veterinarian, and complete blood chemistry panels were routinely performed, without identification of significant abnormalities. By one month of treatment, repeat ultrasonography demonstrated no further growth of the renal mass, that is, stable disease. However, the dog developed a generalized inflammatory condition that involved the skin, eyes, and nasal passages. Skin lesions ranged from nonpruritic maculopapular rash to urticaria, erythema nodosum, and purpura. The oral mucosa was inflamed, and uveitis was prominent. There was also episodic fever and an apparent increase in the patient's arthritis. Because the dog's owner had previously fed him large amounts of vegetable matter rich in phytates, the inflammatory reaction was originally postulated to be due to zinc deficiency caused by phytate sequestering of this critical metal. Zinc deficiency is known to produce symptoms very similar to those observed in this case. However, zinc supplementation did not improve his condition, and alternative causes for the inflammatory reaction were sought.

Based upon our earlier* in vitro* work [6, 7], we considered the possibility that inhibition of the mevalonate pathway might be the cause of the autoinflammatory reaction observed. This proved to be the case. Administration of oral, encapsulated geraniol (60/mg/kg/day) was followed by a rapid clearing (3.5 days) of all lesions. This dog survived 315 days from original diagnosis, with pulmonary metastasis being the cause of death.

*Case 2* is a four-and-one-half-year-old, 28.4 kg, intact female Doberman pinscher who presented with lameness in her rear right leg in November of 2013. At that time, she was diagnosed with decreased conscious proprioception in the right hind limb. The patient was subsequently referred to the Oregon State University College of Veterinary Medicine in October of 2015 after presenting with non-weight bearing lameness and muscle atrophy on the right hind limb. Radiology of the hips revealed periosteal reaction on the right ischiatic table with a mass-like effect in the adjacent musculature. Ultrasound of the musculature adjacent to the right ischium showed an intramuscular mass. A fine needle aspirate of the mass was performed and confirmed a soft tissue sarcoma. Amputation followed by traditional chemotherapy was recommended, which was declined by the owners of this dog. The patient's hepatorenal values were also noted to be consistently elevated, which would potentially have complicated traditional chemotherapy.

**Figure 1:**
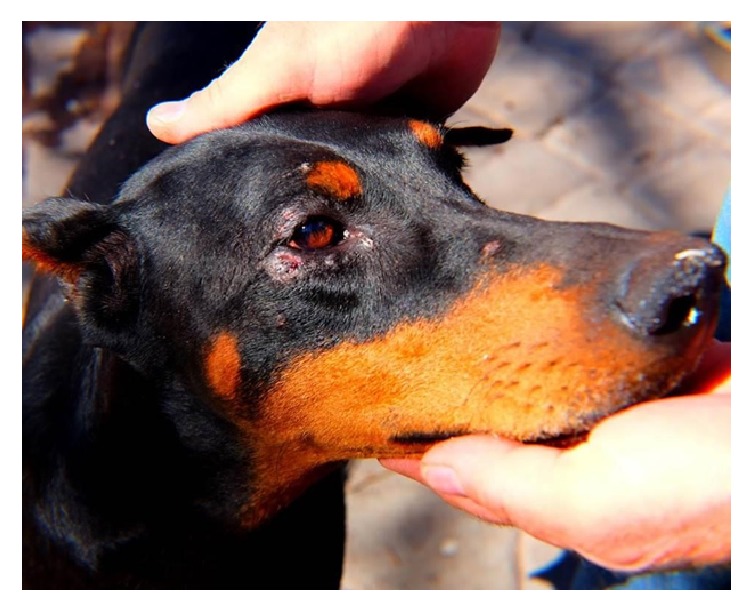
Inflammation in and about the eye in a Doberman treated with G6PD inhibition for a cytologically verified soft tissue sarcoma in her right ischium. Inflammation such as this is a feature of the human autosomal recessive disease mevalonate kinase deficiency, caused by NALP3 inflammasome-mediated IL1*β* secretion [8].

**Figure 2:**
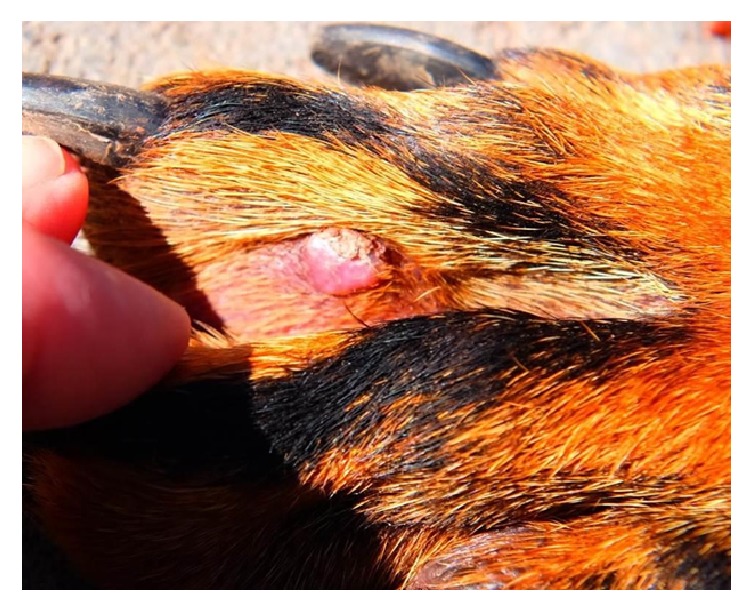
Interdigital inflammatory skin lesion closely resembling those observed in MKD patients. This patient had numerous such lesions on her extremities, trunk, and foot pads.

**Figure 3:**
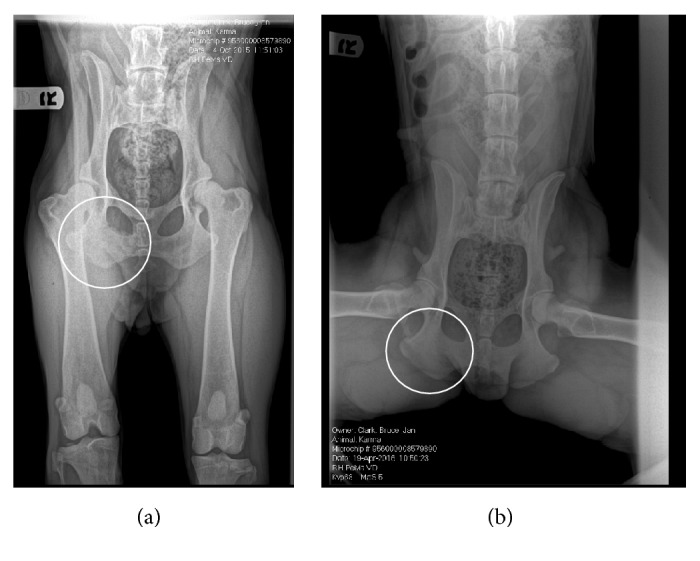
Radiographs of a dog with soft tissue sarcoma before and after treatment with high dose DHEA. Note the right ischial soft tissue sarcoma (a) which completely resolved as a result of treatment (b).

This dog was entered into our G6PD inhibition protocol in October of 2015 and was treated daily with 60 mg/kg/day DHEA and 0.1 mg/kg/day ubiquinone. During treatment, an acute inflammatory reaction involving the eyes was noted (Figure 1). This was followed by multiple inflammatory cutaneous lesions on the legs, foot pads, and trunk (Figure 2). These lesions closely resembled those observed in Case 1. Additionally, this dog had intermittent episodes of fever and an apparent increase in arthralgia. Upon administration of oral geraniol (60 mg/kg/day in divided doses for seven days), all symptoms completely subsided. Of note, posttreatment radiographs showed complete tumor regression (Figure 3). As of May 2017, this patient is alive and well with no evidence of tumor recurrence or recurring inflammation. Incidentally, she became pregnant during high dose DHEA treatment, subsequently delivering five healthy puppies, demonstrating that G6PD inhibition is surprisingly nontoxic to the developing fetus.

Besides the similarity in gross appearance of the skin lesions, histologically both cases showed a neutrophilic exudate, and numerous bacteria (cocci or coccobacilli) were visible in surface keratin and hair follicles. There was a diffuse infiltrate of plasma cells, lymphocytes, neutrophils, macrophages, and eosinophils and, in Case 1, clear hypertrophy of dermal sebaceous and apocrine glands. Additionally, both cases responded to geraniol with clearing of the autoinflammatory lesions. In subsequent studies, it was demonstrated that simultaneous administration of geraniol or other terpenes with DHEA could prevent the formation of such autoinflammatory lesions.

## 3. Discussion

Because of its prominent role in initiating and maintaining the neoplastic state, G6PD is the subject of intense investigation as a potential target for treatment of both human and veterinary cancer. Our work using the uncompetitive G6PD inhibitor DHEA demonstrates that systemic G6PD inhibition in dogs induces a severe autoinflammatory reaction comprising intermittent fever, apparent arthralgia, severe skin rash, and aphthous ulcers of mucocutaneous tissues. These same clinical manifestations appear in the rare autosomal recessive disease in humans called mevalonate kinase deficiency (MKD), including ocular involvement [8]. MKD is apparently caused by interference with the geranylation of the small GTPases Rac and Rho, resulting in activation of the NALP3 inflammasome, and release of interleukin 1*β* [9, 10]. In animal models of MKD, the geranylation of Rac and Rho can be reconstituted by administration of the isoprene precursor geraniol [11]. In* in vitro* studies of inhibition by DHEA of protein geranylation and farnesylation (together referred to as protein isoprenylation), we had demonstrated that the block to protein isoprenylation could be relieved by the addition of the isoprene donor geraniol [6, 7]. The autoinflammatory skin lesions observed in the dogs reported here were also found to be effectively treated with geraniol, and in subsequent studies it has been demonstrated that simultaneous geraniol prevents the autoinflammatory reaction from evolving. These data strongly suggest that, as in MKD, the autoinflammatory reaction observed in dogs treated with high dose DHEA is caused by inhibition of the mevalonate pathway and can be prevented by using geraniol.

It is important to note that the autoinflammatory reaction that we have observed in dogs does not occur in laboratory mice or rats treated with high dose DHEA. Rats and mice have no circulating DHEA sulfate (DHEAS), and the p53-associated “kill-switch” that we postulate to exist in animals with circulating DHEAS appears not to have evolved in them. Dogs, however, do have appreciable levels of circulating DHEAS, albeit not in the range observed in humans and some other primates [12].

## 4. Conclusion

Systemic inhibition of G6PD induces a severe autoinflammatory reaction in dogs, and potentially in other species that have evolved circulating DHEAS, but not in rats and mice. G6PD inhibitors cannot, therefore, be used in canines without the risk of inducing this serious MKD-like autoinflammatory reaction, unless protein geranylation is reconstituted. Preclinical studies of G6PD inhibitors intended for use in nonrodent species, including man, should take this finding into consideration.
